# Supplementary material for: Constructing of Ni-Nx Active Sites in Self-Supported Ni Single-Atom Catalysts for Efficient Reduction of CO2 to CO
Source: Nanomaterials (Basel). 2025 Mar 20;15(6):473. doi: 10.3390/nano15060473 (PMC11946393; doi:10.3390/nano15060473)
Supplement: Supplementary file 1 [file nanomaterials-15-00473-s001.zip › nanomaterials-3513332-supplementary.pdf]

Supporting information

Constructing Ni-Nx active sites in self-supported Ni single-atom catalysts for efficient CO_2 reduction to CO

Xuemei Zhou[1], Chunxia Meng[1], Wanqiang Yu, Yijie Wang, Luyun Cui, Tong Li, Jingang Wang*

Institute for Advanced Interdisciplinary Research (iAIR), School of Chemistry and Chemical Engineering, University of Jinan, Jinan, 250022, P. R. China

*Email: chm_wangjg@ujn.edu.cn (J.G. Wang)

[1] These authors contributed equally to this work.

Experimental Section

Characterization

The morphology and composition of the catalysts were analyzed by scanning electron microscopy (SEM, HITACHI Regulus 8100), X-ray photoelectron spectroscopy (XPS, ESCALAB Xi^{+}), transmission electron microscopy and energy dispersive X-ray spectroscopy (TEM and EDS, JEM-2100F), high-angle annular dark-field scanning transmission electron microscopy (HAADF-STEM, FEI Themis Z), X-ray powder diffraction (XRD, Ultima IV), and Brunauer–Emmett–Teller (BET, KuBo-X1000). X-ray absorption fine structure (XAFS) analysis was carried out at the Shanghai Synchrotron Radiation Center.

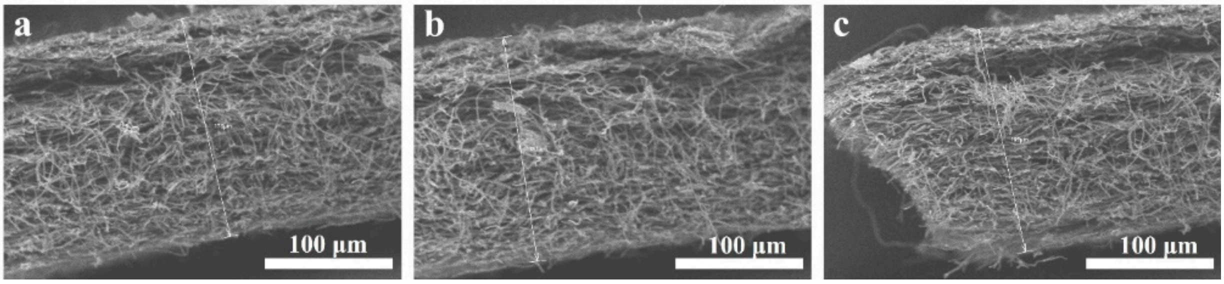

Figure S1 Different cross sections of the same catalyst in SEM and their dimensions

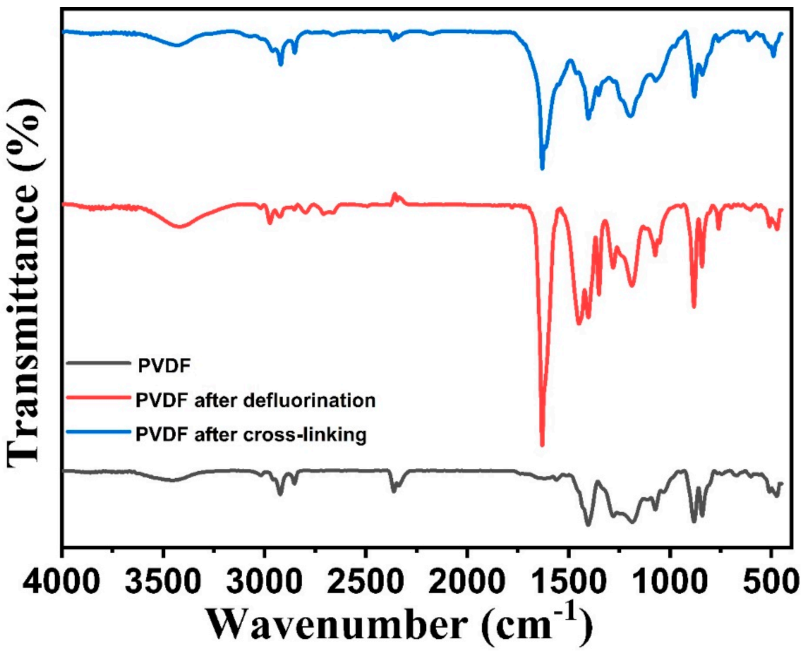

Figure S2. Infrared spectra of PVDF, PVDF after defluorination and PVDF after cross-linking.

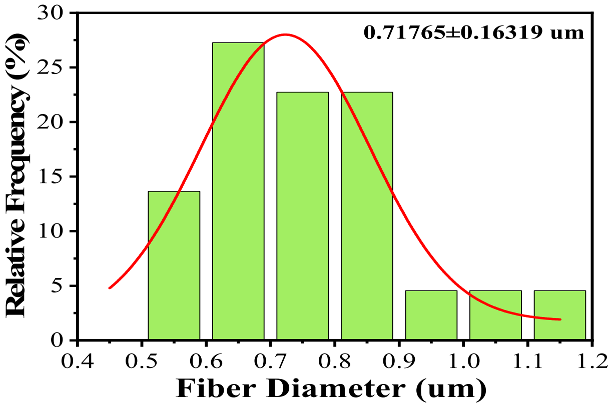

Figure S3. Diameter distribution of Ni-PVDF membrane fibers.

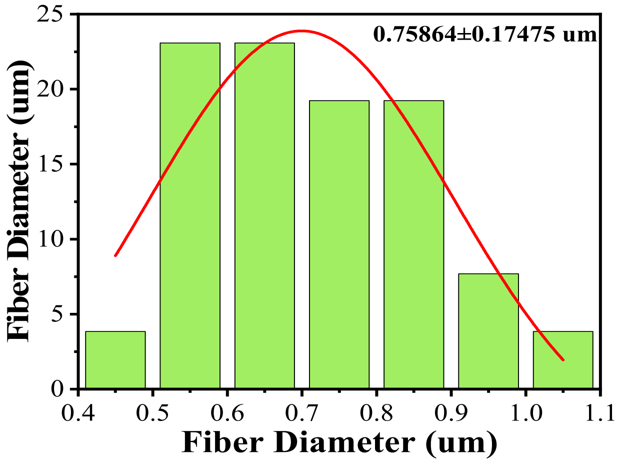

Figure S4. Fiber diameter distribution chart of Ni-PVDF membrane after carbonization.

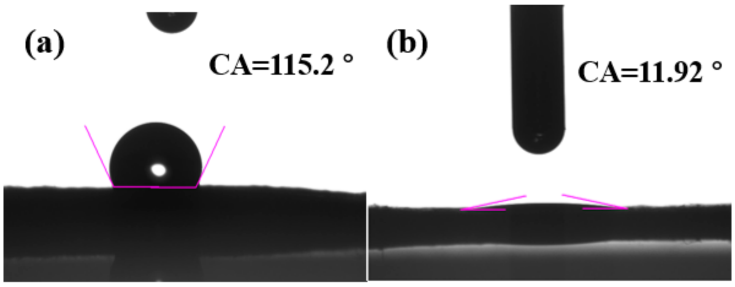

Figure S5. Contact angles of Ni-PVDF membrane before and after carbonization.

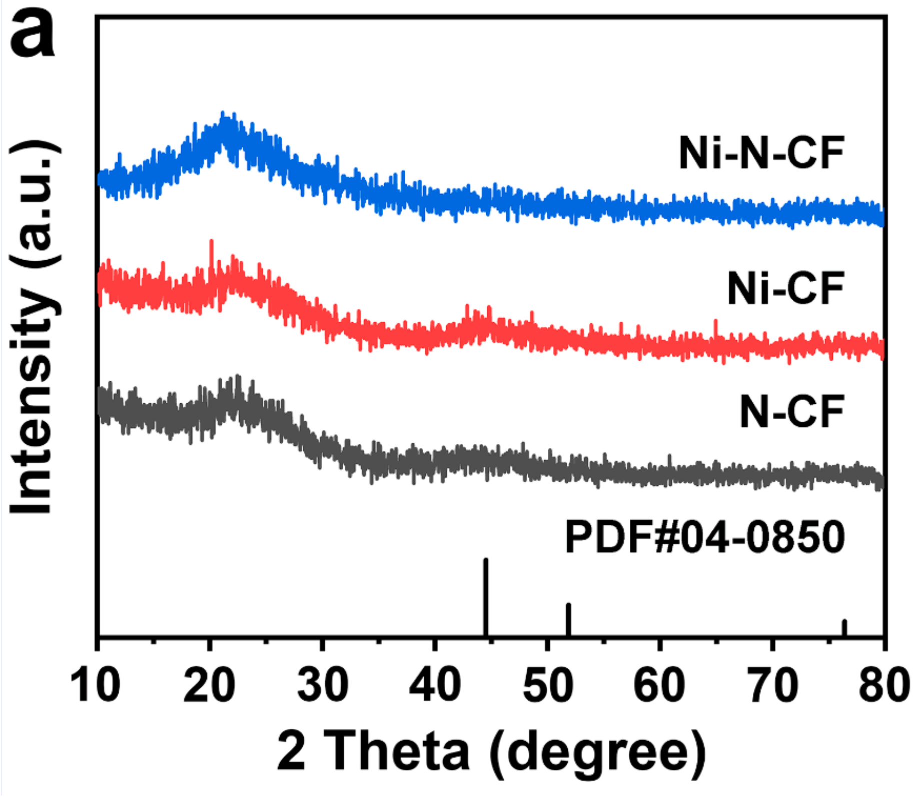

Figure S6 XRD spectra of Ni-N-CF, Ni-CF and N-CF catalyst.

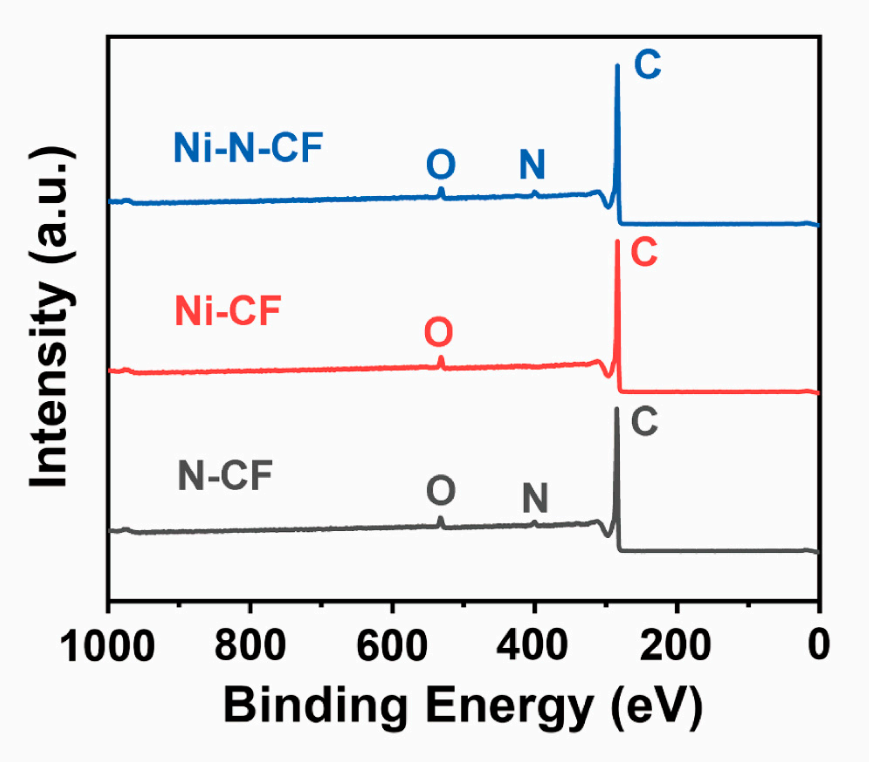

Figure S7 XPS spectra of Ni-N-CF, Ni-CF and N-CF catalyst.

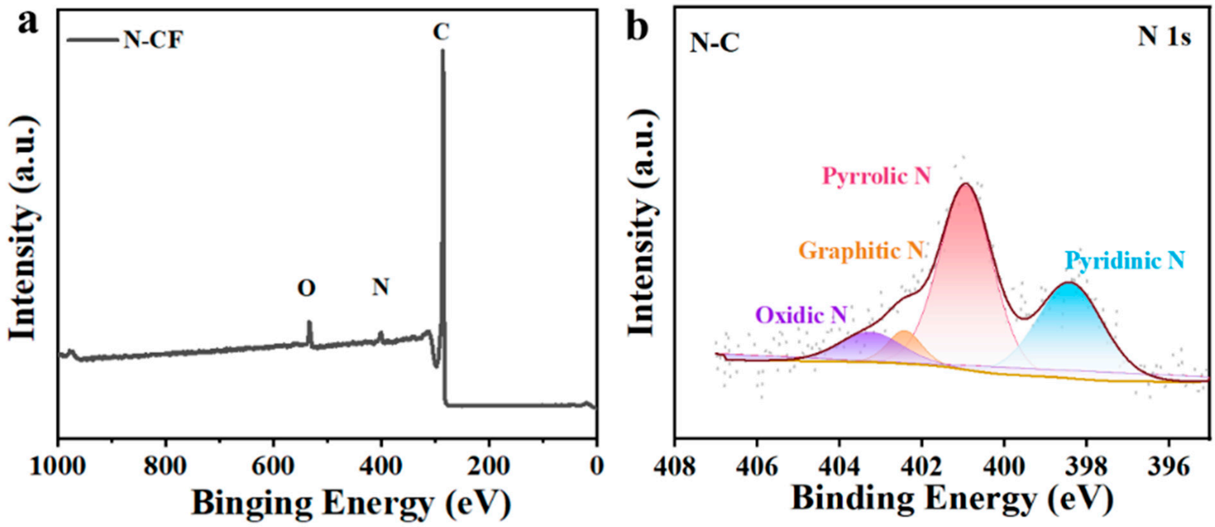

Figure S8. XPS spectra of N-CF. (a) XPS survey spectra. (b) N 1s.

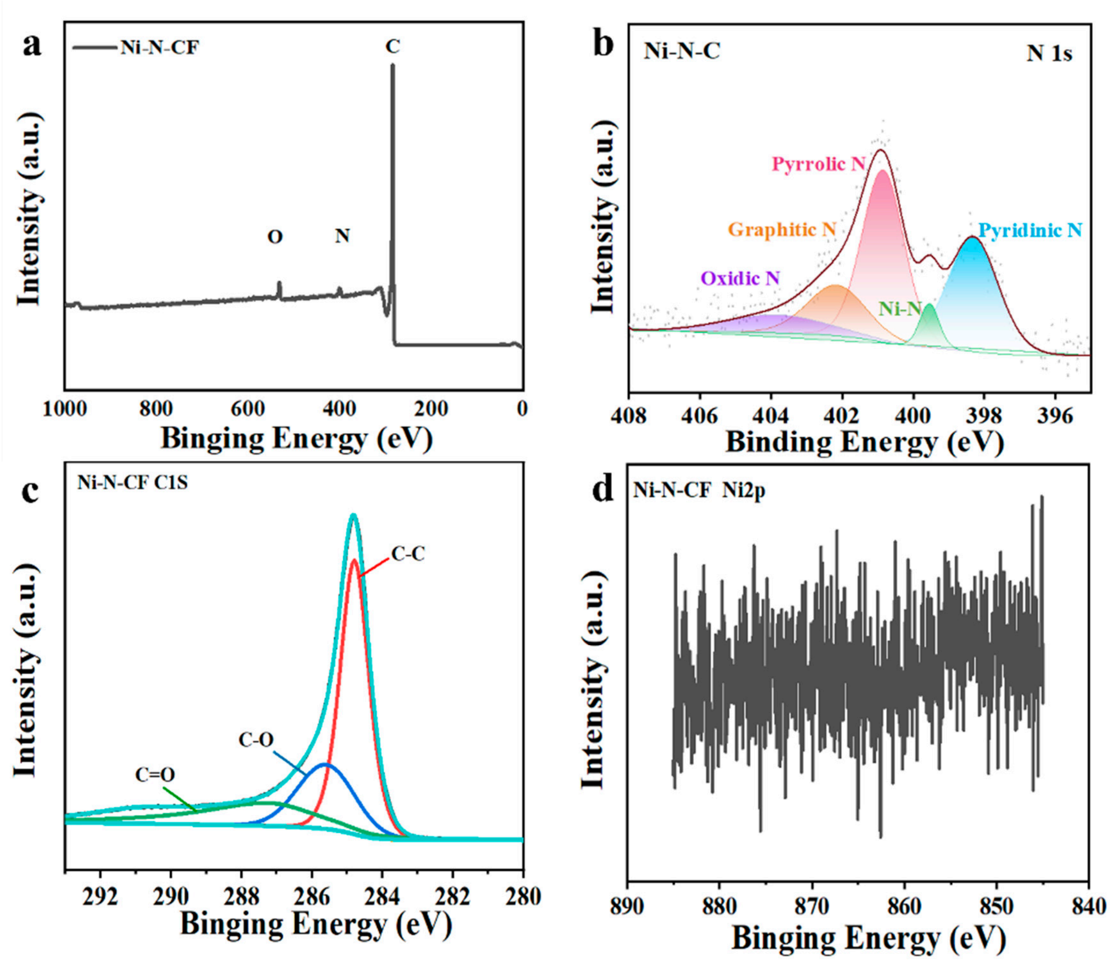

Figure S9. XPS spectra of Ni-N-CF. (a) XPS survey spectra. (b) N 1s. (c) C 1s and (d) Ni 2p.

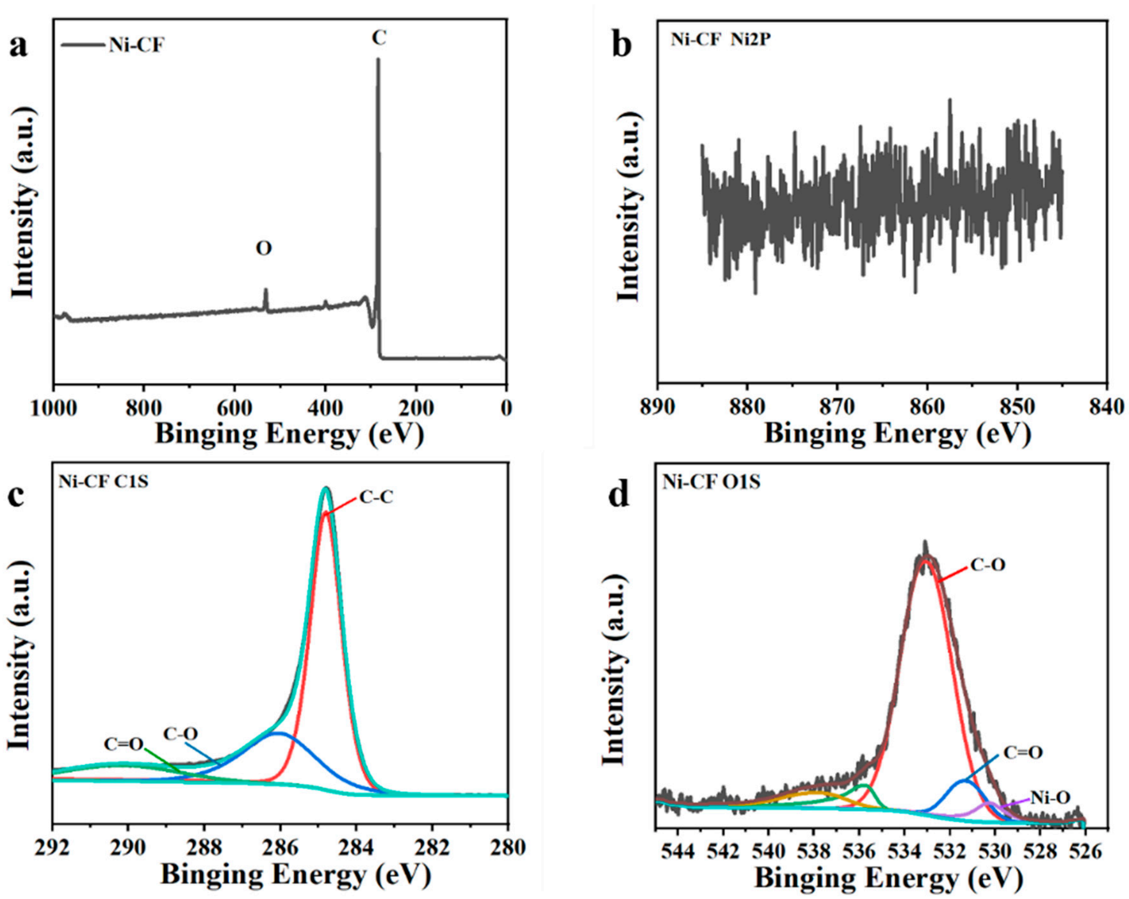

Figure S10. XPS spectra of Ni-CF. (a) XPS survey spectra. (b) Ni 2p. (c) C 1s and (d) O 1s.

Table S1 ICP-MS Data Table of Ni-N-CF

Sample	Sample quality m_0 (g)	Constant volume V_0 (mL)	Test element	Test solution element concentration C_0 (μg/L)	Dilution factor f	Sample element content C_x(μg/kg)	Sample element content W
Ni-N-CF	0.0049	25	Ni	156.326	1	797581.633	0.080

Table S2 Comparison of recent Ni-N_4 electrocatalysts for CO_2RR to CO.

Samples	Electrolyte	FE $_{formate}$	Potential vs. RHE (V)	j_{CO} (mA cm^{-2})	Ref.
Ni-N-CF	0.5 M $NaHCO_3$	92%	−0.65	2.8	This work
Ni SAs/N-C	0.5 M $KHCO_3$	71.9%	−0.89	10.48	[39]
TPPDA-NiPor-COF	0.5 M $KHCO_3$	76%	−0.9	5.05	[57]
Ni-N-C	0.5 M $KHCO_3$	80%	−0.49	20	[58]
Cu_1Zn_9–Ni	0.5M $KHCO_3$	80%	−0.8	9	[59]
Ni-N-C-rGO	0.5 M $KHCO_3$	85%	−0.87	10	[60]
3D-CE-NiFe	0.1M $KHCO_3$	87.80%	−0.7	1.8	[61]
$Ni(OH)_2$@RGO	0.1 M $KHCO_3$	88.2%	−0.9	2.3	[62]
Ni-N-MEGO	0.5M $KHCO_3$	89%	−0.55	26.8	[63]

Reference:

[39] Zhao, C.-M.; Dai, X.-Y.; Yao, T.; Chen, W.-X.; Wang, X.-Q.; Wang, J.; Yang, J.; Wei, S.-Q.; Wu, Y.; Li, Y.-D. Ionic exchange of metal−organic frameworks to access single nickel sites for efficient electroreduction of CO_2. *J. Am. Chem. Soc*. **2017**, *139,* 8078−8081.

[57] Gong, L.; Chen, B.-T.; Gao, Y.; Yu, B.-Q.; Wang, Y.-H.; Han, B.; Lin, C.-X.; Bian, Y.-Z.; Qi, D.-D.; Jiang, J.-Z. Covalent organic frameworks based on tetraphenyl-p-phenylenediamine and metalloporphyrin for electrochemical conversion of CO_2 to CO. *Inorg. Chem*. **2022**, *9,* 3217–3223.

[58] Lyu, X.; Anastasiadou, D.; Raj, J.; Wu, J.-J.; Bai, Y.-C.; Li, J.-L.; Cullen, D.-A.; Yang, J.; Gonçalves, L.-P.-L.; Lebedev, O.-I.; Yury V. Kolen, Y.-V.-K.; Figueiredo, M.-C.; Serov, A.; Large-scale synthesis of metal/nitrogen Co-doped carbon catalysts for CO_2 electroreduction. *Electrochim. Acta.* **2023**,*255*, 142427.

[59] Chen, J.-X.; Wei, X.-F.; Cai, R.-M.; Ren, J.-Z.; Ju, M.; Lu, X.-Q.; Long, X.; Yang, S.-H. Composition-Tuned surface binding on CuZn-Ni catalysts boosts CO_2RR selectivity toward CO generation. *ACS Materials Lett.* **2022**, *4,* 497−504.

[60] Wang, F.-Y.; Liu, Y.; Song, Z.-L.; Miao, Z.-C.; Zhao, J.-P. Ni-N-Doped carbon-modified reduced graphene oxide catalysts for electrochemical CO_2 reduction reaction. *Catalysts.* **2021**, *11*, 561.

[61]. Sheng, J.-Y.; Gao, M.-S.; Zhao, N.; Zhao, K.; Shi, Y.-A.; Wang, W. Bimetallic Ni/Fe functionalized, 3D printed, self-supporting catalytic-electrodes for CO_2 reduction reaction. *FUEL.* **2025**, *382*, 133703.

[62]. Liu, E.; Liu, T.-X.; Ma, X.-J.; Zhang, Y.-P. The electrocatalytic performance of Ni–$AlO(OH)_3$@RGO for the reduction of CO_2 to CO. *New J. Chem.* **2022**, *46*, 12023.

[63]. Cheng, Y.; Zhao, S.-Y.; Li, H.-B.; He, S.; Veder, J.-P.; Johannessen, B.; Xiao, J.-P.; Lu, S.-F.; Pan, J.; Chisholm, M.-F.; Yang, S.-Z.; Liu, C.; G. Chen, J.-G.; Jiang, S.-P. Unsaturated edge-anchored Ni single atoms on porous microwave exfoliated graphene oxide for electrochemical CO_2. *ACB-Env.* **2019**, *243*, 294-303.
